# Resting state functional connectivity as a marker of internalizing disorder onset in high-risk youth

**DOI:** 10.1038/s41598-022-25805-y

**Published:** 2022-12-09

**Authors:** McKinley Pawlak, Signe Bray, Daniel C. Kopala-Sibley

**Affiliations:** 1 Hotchkiss Brain Institute, University of Calgary, Calgary, AB Canada; 2 Alberta Children Hospital Research Institute (ACHRI), University of Calgary, Calgary, AB Canada; 3 Mathison Centre for Mental Health Research and Education, University of Calgary, Calgary, AB Canada; 4 Child and Adolescent Imaging Research (CAIR) Program, University of Calgary, Calgary, AB Canada; 5 Department of Radiology, University of Calgary, Calgary, AB Canada; 6 Department of Pediatrics, University of Calgary, Calgary, AB Canada; 7 Department of Psychiatry, University of Calgary, Calgary, AB Canada

**Keywords:** Cognitive neuroscience, Emotion, Functional magnetic resonance imaging, Human behaviour, Predictive markers, Anxiety, Depression

## Abstract

While research has linked alterations in functional connectivity of the default mode (DMN), cognitive control (CCN), and salience networks (SN) to depression and anxiety, little research has examined whether these alterations may be premorbid vulnerabilities. This study examined resting state functional connectivity (RSFC) of the CCN, DMN, and SN as markers of risk for developing an onset of a depressive or anxiety disorder in adolescents at high familial risk for these disorders. At baseline, 135 participants aged 11–17 completed resting-state functional magnetic resonance imaging, measures of internalizing symptoms, and diagnostic interviews to assess history of depressive and anxiety disorders. Diagnostic assessments were completed again at 9- or 18-month follow-up for 112 participants. At baseline, increased CCN connectivity to areas of the visual network, and decreased connectivity between the left SN and the precentral gyrus, predicted an increased likelihood of a new onset at follow-up. Increased connectivity between the right SN and postcentral gyrus at baseline predicted first episode onsets at follow-up. Altered connectivity between these regions may represent a risk factor for developing a clinically significant onset of an internalizing disorder. Results may have implications for understanding the neural bases of internalizing disorders for early identification and prevention efforts.

## Introduction

Rates of depression and anxiety increase sharply in adolescence, especially in females where instances of first episode depression or anxiety are approximately double that of males^1^. A single episode of depression or anxiety can lead to enduring negative psychosocial consequences, such as suicidality, addiction, and unemployment^2^. In addition, an episode can leave the individual vulnerable to further episodes, at which point depression and anxiety can become chronic, treatment-resistant disorders^3^.

Recent neuroimaging research in internalizing disorders (i.e., depression and anxiety) has aimed to find neural markers of risk in youth, but there is a lack of longitudinal research predicting future episode onset in high-risk youth^4,5^. This study aims to investigate if functional connectivity between core intrinsic networks previously implicated in internalizing psychopathology could act as a neural marker of risk for internalizing disorder onsets in a longitudinal cohort of youth at high risk, by virtue of having a parent with a history of these disorders. Resting state functional connectivity (RSFC) is assessed via the temporal correlation of blood oxygenation level dependent (BOLD) signal in anatomically separate brain regions while the participant rests passively in the scanner^6^. Several reviews and meta-analyses have found altered RSFC in depression and anxiety, particularly in the DMN, CCN, and SN resting state networks^7–10^. Determining potential neural markers of the onset of internalizing disorders in high-risk youth is important for developing and implementing early intervention strategies to reduce illness severity or even prevent onset of the disorder.

### Familial risk for depression and anxiety

Anxiety and depression both run in families, with genetic heredity of risk factors, as well as parenting and environmental effects interacting in high familial risk (subsequently “high-risk”) populations to increase risk for developing the disorder to 3–5 times that of low-risk populations^11,12^. High-risk adolescents show similar cognitive risk factors as depressed adults, with negative cognitive styles and biases in attention, interpretation, and memory of emotionally-valenced experiences^11^. Depression and anxiety both have similar hallmark characteristics such as increased rumination and negative self-thought, cognitive biases, and cognitive processing problems, as well as emotional control deficits^8,10^. These cognitive features have been linked to alterations in within-network functional connectivity of three intrinsic connectivity networks, the default mode network (DMN), the cognitive control network (CCN), and the salience network (SN)^7–10^.

### Intrinsic resting-state brain networks and internalizing psychopathology

The DMN is comprised of the posterior cingulate cortex (PCC)/precuneus, middle and lateral parietal and temporal cortices, and medial prefrontal cortex (PFC)^13^. The DMN is de-activated during tasks requiring external attention, relative to passive rest, and is involved in self-referential processing, internal thought, and mental simulation, core processes that are altered in depression and anxiety^13^. In a study of individuals at high-familial risk for major depressive disorder (MDD) it was found that high- versus low-risk participants had increased DMN RSFC^14^. Increased RSFC of the DMN in patients with MDD has been shown to reflect increased negative self-thought and maladaptive rumination^15,16^. However, some studies have found decreased functional connectivity in individuals with high-trait anxiety, subthreshold depression, and MDD, indicating that while DMN RSFC is related to depression and anxiety the direction of effects are inconsistent^17–19^.

The cognitive control network (CCN) is a series of brain regions that are functionally coupled during performance of working memory, inhibitory control, selective attention, cognitive flexibility, and fluid reasoning^20^. It includes the anterior cingulate cortex (ACC)/pre-supplementary motor area, inferior frontal junction, anterior insular (AI) cortex, dorsolateral prefrontal cortex (DLPFC), dorsal pre-motor cortex, and posterior parietal cortex^21^. Altered functional connectivity of cognitive control regions such as the DLPFC and regions in the posterior parietal cortex are associated with cognitive control impairments and cognitive biases in depression^22,23^. The direction of reported RSFC abnormalities in the CCN in depression and anxiety is variable^24^. Some studies in high-risk populations as well as those with subthreshold depression or elevated trait anxiety have found reduced CCN RSFC^17,22,25–27^. Others found increased CCN connectivity in those with versus without anxiety disorders^28^. Still more have found both increased and decreased RSFC within the CCN when looking at connectivity between discrete regions^24,29^. Overall, while alterations of CCN RSFC are seen consistently across depression and anxiety, the direction is highly variable.

The salience network (SN) includes the amygdala, dorsal striatum, dorsal and subgenual ACC, and the AI and is involved in detection and processing of salient stimuli both internally and externally as well as recruiting necessary functional networks in response to said stimuli^30^. One of the primary roles of the SN is to control switching from the DMN to the CCN based on salience of external vs internal stimuli and thus may play a role in inter-network dysfunction in depression and anxiety where there can be a bias towards internal stimuli or excessive attention to perceived threatening stimuli^16,28–30^. In adolescent females increased within-SN RSFC was associated with greater subclinical anxiety and depressive symptoms^31^. Individuals with depressive and anxiety disorders, relative to those without, show impairments in the salience processing of emotional and threatening stimuli, often overattributing salience to neutral stimuli^30^. The SN is one of the least studied RSFC networks in depression and anxiety and as such the direction of alterations in RSFC in depression and anxiety is still to be elucidated.

FC of these networks has been linked to subclinical depressive and anxiety symptoms and may differentiate those with versus without clinically significant depression or anxiety. However, the direction of association is inconsistent, and it is unknown if alterations in these networks are a result of the disorders or exist prior to the onset of the disorder and confer vulnerability to their development^4,5^. As such these networks’ RSFC may act as markers of the onset of depression and anxiety in at-risk youth. Testing this possibility requires a sample of adolescents at high risk for these disorders but who have yet to develop them prior to neuroimaging.

### Overview and objectives

There is limited longitudinal research in adolescents at high familial risk for depression and anxiety predicting episode onset as a function of functional connectivity in the DMN, CCN, and SN resting state networks^5^. The first aim of this study was therefore to examine whether whole brain RSFC of the CCN, DMN, and SN represent possible neural markers of risk for new onsets of internalizing disorders in adolescents at high familial risk. As a secondary aim, this study examined whether RSFC of these networks predicts first lifetime onsets of internalizing disorders in adolescents. This will aid in determining if the altered RSFC seen in depressed or anxious adults represents a risk factor for future internalizing disorder onsets as well as a premorbid vulnerability factor for depression and anxiety in high-risk teens. We hypothesize that altered functional connectivity between the CCN, DMN, SN, and the rest of the brain will longitudinally predict onsets of a diagnosable episode of an internalizing disorder. It is important to understand alterations in RSFC in different stages of illness, particularly in at-risk youth, as associations of these alterations with internalizing disorders may vary depending on disorder stage.

## Methods

### Participants

Participants were 135 adolescents and pre-adolescents ages 11–17. This age range was chosen as it precedes and includes the developmental period in which rates of depression and anxiety in youth increase, and female risk doubles compared to males^1^. Data were drawn from the Calgary Biopsychosocial Risk for Adolescent Internalizing Disorders (C-BRAID) study. Participants were recruited based on parental history of either a depressive or anxiety disorder, without having experienced an episode themselves. This risk-enriched design increased the likelihood of participants developing depressive or anxiety disorder episodes during the study period. Additional exclusion criteria included contraindications for MRI, history of traumatic brain injury, and presence of a developmental disorder. This study was approved, and data was collected in accordance with the institutional Research and Ethics Board, the Conjoint Health Research Ethics Board (CHREB) at the University of Calgary. All methods were performed in accordance with the relevant guidelines and regulations, and informed consent was obtained from participants or their legal guardian, minors additionally provided assent.

Demographic characteristics are shown in Table 1. Adolescents were 60% female (*n* = 81), with a mean age of 13.70 (standard deviation = 1.53), and 66.7% identified as white/Caucasian (*n* = 90). Parents were 92.6% female (*n* = 125), with a mean age of 43.15 (standard deviation = 6.07), and 78.5% identified as white/Caucasian (*n* = 106). Most parents were married or in a common-law relationship (71.9%, *n* = 97), with 50.4% having a college/university education or higher (*n* = 68), and the median annual household income was approximately $87,500. Participant demographics are approximately representative of demographics in the local catchment area. At baseline 48 parents had a lifetime occurrence of a depressive disorder, 7 had a lifetime occurrence of an anxiety disorder, and 73 having had an occurrence of both depression and anxiety in their lifetime. At baseline 5 children had a lifetime occurrence of depression, 5 had a lifetime occurrence of anxiety, and 2 had an occurrence of both depression and anxiety in their lifetimes (Table 2). This was due to discrepancies in answers between screening and interviews and was potentially driven by lack of privacy during screening phone calls, where adolescents may have been unwilling to disclose mental health information over the phone in the presence of their parents.

**Table 1:** Sociodemographic characteristics.

Characteristic	Parent	Child
Mean age (SD)	43.15 (6.07)	13.70 (1.53)
**Sex**		
Female	125	81
Male	10	54
**Ethnicity**		
Caucasian/White	106	90
Asian/Pacific Islander	12	9
Indigenous	4	4
Hispanic/Latino	7	3
Arabic	2	2
Multiracial	3	24
Other	1	2
Did not disclose	0	1
**Marital status**		
Single/never married	9	
Married/common law	97	
Divorced/separated	29	
**Annual household income (CAD)**		
< $25,000	8	
$25–50,000	22	
$50–75,000	14	
$75–100,000	24	
$100–125,000	20	
$125–150,000	11	
$150–175,000	9	
< $175,000	21	
Did not disclose	6	
**Education**		
Some High School	6	
High School Diploma	6	
Some College/University	32	
Trade School	22	
College Degree or more	68	
Other	3	

*SD* standard deviation, *CAD* Canadian dollar.

**Table 2:** Parent and child internalizing disorders diagnoses.

	Parent		Child		
	Current	Lifetime	Baseline	9-Month	18-Month
Major depressive disorder	17	103	6	4	12
Persistent depressive disorder	8	22	0	0	1
Bipolar disorder	–	15	1	1	0
Generalized anxiety disorder	27	38	1	2	3
Social anxiety disorder	10	26	4	2	1
Panic disorder	19	44	3	2	1
Separation anxiety	–	–	0	0	0

Breakdown of past diagnoses at baseline and new onsets at follow-up in children and parent current and lifetime diagnoses. There is overlap between diagnoses.

Baseline rs-fMRI data was available from 135 participants, and 130 completed baseline internalizing symptom measures. Out of that 130, 88 completed diagnostic interviews at 9-month follows-ups to assess for DSM-V criteria for an internalizing disorder^32^. 77 completed 18-month follow-up diagnostic interviews. 53 have completed both 9-month and 18-month follow-ups. In total, 112 have completed at least one follow-up diagnostic interview as well as baseline MRI scans. Our effective sample size for new onsets is therefore 112 adolescents. Little’s Missing Completely at Random (MCAR) test including all variables in the current study was non-significant (Chi-Square = 9.635, df = 12, *p* = 0.648), indicating data are missing at random and participants with missing data do not differ significantly from those with complete data on any study variable^33^. Participants with missing data but had completed at least one diagnostic assessment at either 9 or 18 months, were therefore included in this analysis (*n* = 112).

When excluding children with a history of depression or anxiety, baseline rs-fMRI data was available from 123 participants, and 119 completed baseline internalizing symptom measures. Out of that 119, 77 completed diagnostic interviews at 9-month follows-ups to assess for DSM-V criteria for an internalizing disorder^32^. 68 completed 18-month follow-up diagnostic interviews. 46 have completed both 9-month and 18-month follow-ups. In total, 99 have completed at least one follow-up diagnostic interview as well as baseline MRI scans. Our effective sample size for first lifetime onsets is therefore 99 adolescents. Little’s Missing Completely at Random (MCAR) test including all variables in the current study was non-significant (Chi-Square = 5.756, df = 12, *p* = 0.928), indicating data are missing at random and participants with missing data do not differ significantly from those with complete data on any study variable^33^. Participants with missing data but had completed at least one diagnostic assessment at either 9 or 18 months were therefore included in this analysis (*n* = 99).

### Procedure

Parent and participant history of depressive or anxiety disorders was assessed via structured diagnostic interview. Only those youth with a parent with a history of major depressive disorder (MDD), persistent depressive disorder (PDD), bipolar disorder (BD), or general anxiety disorder (GAD) or social anxiety disorder (SAD), but who have not yet met DSM-V criteria for any of these illnesses themselves were included (see Table 2 for parent diagnoses)^32^. Adolescents completed self-reports of internalizing (i.e., global depressive and anxiety) symptoms at baseline^34^. Adolescents also completed rs-fMRI resting state scans at baseline. Adolescent participants completed structured diagnostic interviews at 9- and/or 18-month follow-ups.

### Measures

#### Mini-international neuropsychiatric interview (MINI)

The MINI was used to assess parents’ lifetime history of either MDD, PDD, BD, GAD or SAD in parents^35^. The MINI-Kid, validated for ages 6–18, was used to confirm no lifetime history of clinically significant internalizing disorders within the youth participants^36^. These brief structured diagnostic interviews show good interrater reliability and respectively converge well with the Structured Clinical Interview for the DSM and the Kiddie Schedule for Affective Disorders (KSADS)^32,37^. The MINI and MINI-Kid were administered by a single interviewer who underwent training by a licensed clinical psychologist. This training included didactic lessons on diagnostic interviewing for mental health, role play exercises, and observation with feedback of the interviewer by the psychologist of interviews with participants. At follow-up assessments, participants are asked about the period since their last visit to the lab. Youth were categorized as having an internalizing disorder over the follow-up period if they met criteria for MDD, PDD, BD, SAD, panic disorder, GAD, or separation anxiety.

#### Youth self-report

The YSR is a 112-item self-report questionnaire for children and adolescents ages 11–18 to assess internalizing and externalizing symptoms^34^. It is a widely used, well validated, self-report measure of depression and anxiety in youth^34^. Behaviors are rated on a 3-point scale: 0-Not true, 1-Somewhat or sometimes true and 2-Very true or often true, based on the preceding 6-months. The current study focuses on the Internalizing Problems subscale which assesses global depressive and anxiety symptoms^32^. This scale shows good internal consistency and respective convergence with interview-based symptom measures of anxiety and depression in adolescents aged 11 to 18^38,39^. Cronbach alpha was 0.89 for our sample.

### Image acquisition and preprocessing

Neuroimaging data were acquired on a GE 3 T 750 MRI. A 12 channel radiofrequency head coil was used with foam padding to restrain head movement. High-resolution T1-weighted 3D BRAVO anatomical volumes [repetition time (TR) = 7.90 ms, echo time (TE) = 3.06 ms, field of view (FOV) = 24 cm, flip angle = 15°, 180 sagittal slices, 1 mm isotropic voxels], and T2* functional images (2D Gradient Echo Planar Imaging) [TR = 2000 ms, TE = 30 ms, FOV = 25.6 cm, flip angle = 75°, 210 axial slices, 4 mm isotropic voxels] were acquired. Rs-fMRI data was acquired in one 7-min scanning session during which participants were told to relax, keep their eyes open and on the fixation cross, and let their mind wander.

Image preprocessing and denoising was done using CONN, a MATLAB (R2020b; MathWorks) toolbox^40^. Functional images were co-registered and resampled to a reference image (first scan of session) using SPM12’s *realign & unwarp* procedure followed by slice timing correction using SPM12’s slice-timing correction (STC) procedure. Functional data was normalized to MNI space and resampled to 2 mm isotropic voxels using SPM12’s unified segmentation and normalization procedure. Outlier identification flagged outliers with framewise displacement greater than 0.2 mm^41^. Framewise displacement was computed by estimating the largest displacement of six control points that are placed at the centre of each side of a bounding box (140 × 180 × 115 mm). Linear regression of confounds included white matter (5 components), cerebrospinal fluid (5 component), motion realignment (12 components; 6 head motion parameters and first order temporal derivatives), scrubbing of any identified outlier scans. Data underwent linear detrending and were bandpass filtered (0.01–0.08) to remove low frequency noise and high frequency physiological noise. Data was smoothed to a Gaussian kernel of 8 mm FWHM. All participants had usable baseline rs-fMRI data, based on having a full 7-min scan (210 slices) and corresponding structural data (180 slices), and a framewise displacement of less than 0.2 mm.

### Statistical analysis

#### Seeds

**Figure 1:**
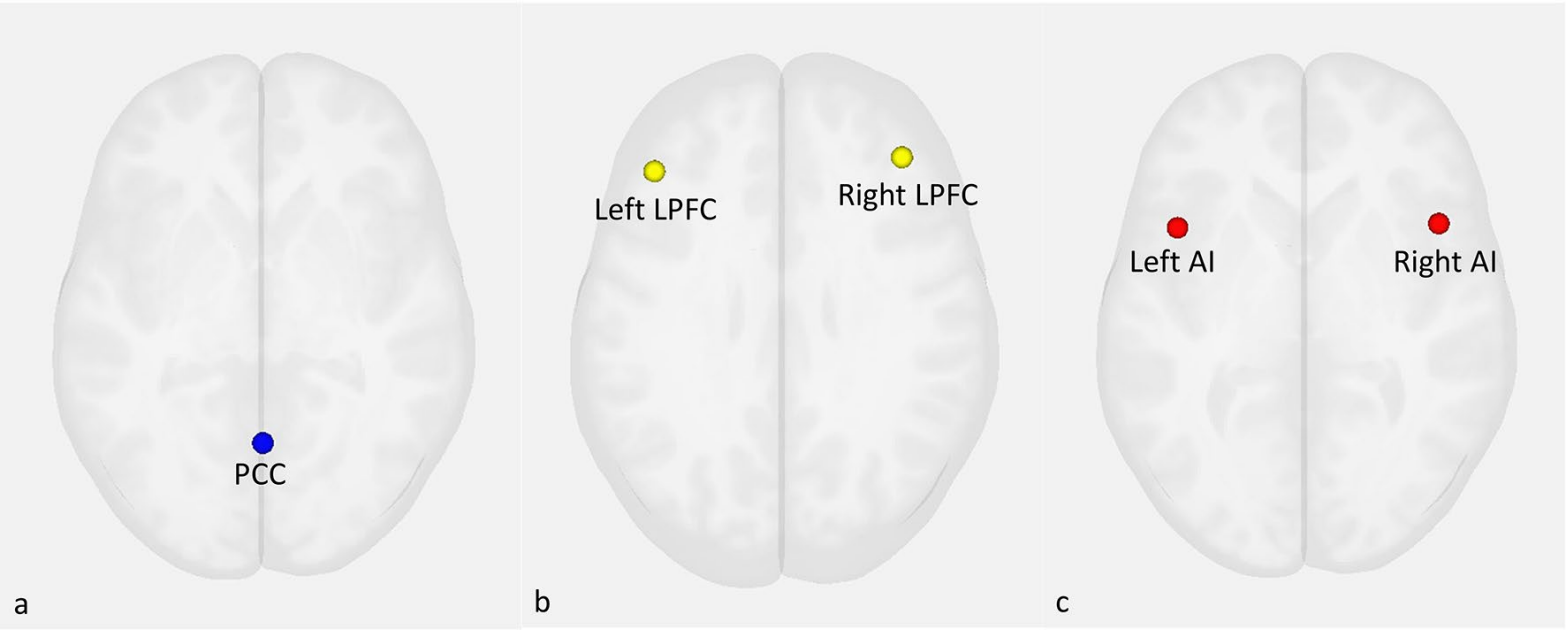
Seed locations for core resting state networks (**a**) default mode network (DMN) seed the posterior cingulate cortex (PCC), (**b**) cognitive control network (CCN) seed the lateral prefrontal cortex (LPFC), (**c**) salience network (SN) seed the anterior insula (AI). Based on the CONN toolbox network regions of interest for MATLAB^40^.

This study calculated FC as seed to whole-brain temporal correlations using seed regions from the CONN toolbox network regions of interest, which were developed from an independent components analysis of the Human Connectome Project (*n* = 497)^40^. Center coordinates were chosen, and 5 mm spherical seeds generated around centers. The DMN (Fig. 1a) region was the PCC (center coordinates: 1, − 61, 38). CCN (Fig. 1b) regions included the bilateral LPFC (L, center coordinates: − 43, 33, 28; R, center coordinates: 41, 38, 30). SN (Fig. 1c) regions included the bilateral AI (L, center coordinates: − 44, 13, 1; R, center coordinates: 47, 14, 0). These regions were chosen as seeds since they have been reliably identified as core regions of each network in previous literature^13,19,21,24,30,42–48^. In the current sample, these seeds elicited functional connectivity in canonical regions associated with each seed’s respective network (Supplementary Tables 1–5, Supplementary Fig. 1).

#### Functional connectivity analyses

First level analysis was done to perform spatial statistical analysis of each participant using CONN. Functional connectivity was determined using a general linear model to determine significant BOLD signal bivariate correlations with respect to time between each seed and each voxel. CONN converted the resulting correlation coefficients to z-scores using Fisher’s Z transformation.

Analyses subsequently consisted of two multiple regression models in CONN, one for new onsets and one for first onsets. The outcome variable was seed to voxel resting state functional connectivity. The predictor variable was whether participants had experienced a DSM-V internalizing disorder (i.e., depressive or anxiety disorder) at either 9-month or 18-month follow-up, based on Diagnostic and Statistical Manual of Mental Disorders (DSM-V) criteria^32^. Five separate seeds (one DMN; two CCN; two SN) were tested. We report results with baseline global internalizing symptoms as a covariate to determine whether any effects of RSFC on disorder onset are over and above baseline subclinical symptoms. This is important given that subthreshold symptoms are the most robust predictor of the onset of future diagnosable illnesses^49^. These analyses are therefore highly conservative. Each seed predictor was analyzed separately with age, sex, and baseline internalizing symptoms as covariates. When examining new onsets, we included participants with a history of depression or anxiety as any onsets at follow-up would be a new episode, in this model we additionally controlled for history of depression or anxiety as a covariate. When examining first episode onsets participants with a history of depression or anxiety at baseline were excluded from analysis.

All functional connectivity whole brain maps at the group level were thresholded using a cluster defining threshold (i.e., voxel-wise threshold) of *p* < 0.001 and a false discovery rate cluster (FDRc) correction of *p* < 0.05, as implemented in CONN. Using this correction method based on Gaussian random field theory, the FDR corrected significance of each cluster is obtained and can be further corrected (e.g., with a Bonferroni correction) for the number of seeds tested. We thus performed a Bonferroni correction based on five seed regions, therefore correcting for 5 comparisons in total. This resulted in a cluster size *p*-threshold of 0.01 (0.05/5 = 0.01).

## Results

### Study participants

#### Descriptive statistics and bivariate correlations

Table 3 includes descriptive statistics and bivariate correlations between the model covariates. Out of our effective sample size of 112, 11 developed only a new onset of a depressive disorder (i.e., MDD, PDD, or BD), 2 developed only a new onset of an anxiety disorder (GAD, SAD, panic disorder, separation anxiety), and 11 developed new onsets of comorbid depressive and anxiety disorders (see Table 2 for breakdown). Thus, a total of 24 out of 112 youth developed new onsets of a DSM-V internalizing disorder at follow-up. When excluding participants with a history of internalizing disorders there were 10 first onsets of only depression, 2 first onsets of only anxiety, and 6 first onsets of comorbid depression and anxiety. Thus, a total of 18 out of 99 youth developed a first episode DSM-V internalizing disorder at follow-up. Females showed increased baseline depressive symptoms and an increased likelihood of an internalizing disorder onset at follow-up (Table 3).

**Table 3:** Descriptive statistics and bivariate correlations of covariates in models.

	Mean	SD	1	2	3	4
Sex (1)						
Age (2)	13.65	1.53	0.168			
Baseline YSR internalizing (3)	13.09	9.71	− 0.284**	0.013		
Baseline diagnosis (4)			− 0.140	0.178*	0.414***	
Follow-up diagnosis (5)			− 0.197*	0.057	0.359***	0.294**

*SD* standard deviation.

*Correlation significant at the p ≤ 0.05 (two-tailed).

**Correlation significant at the p ≤ 0.01 (two-tailed).

***Correlation significant at the p ≤ 0.001 (two-tailed).

### Predicting new diagnosable onset of DSM-V internalizing disorder

**Figure 2:**
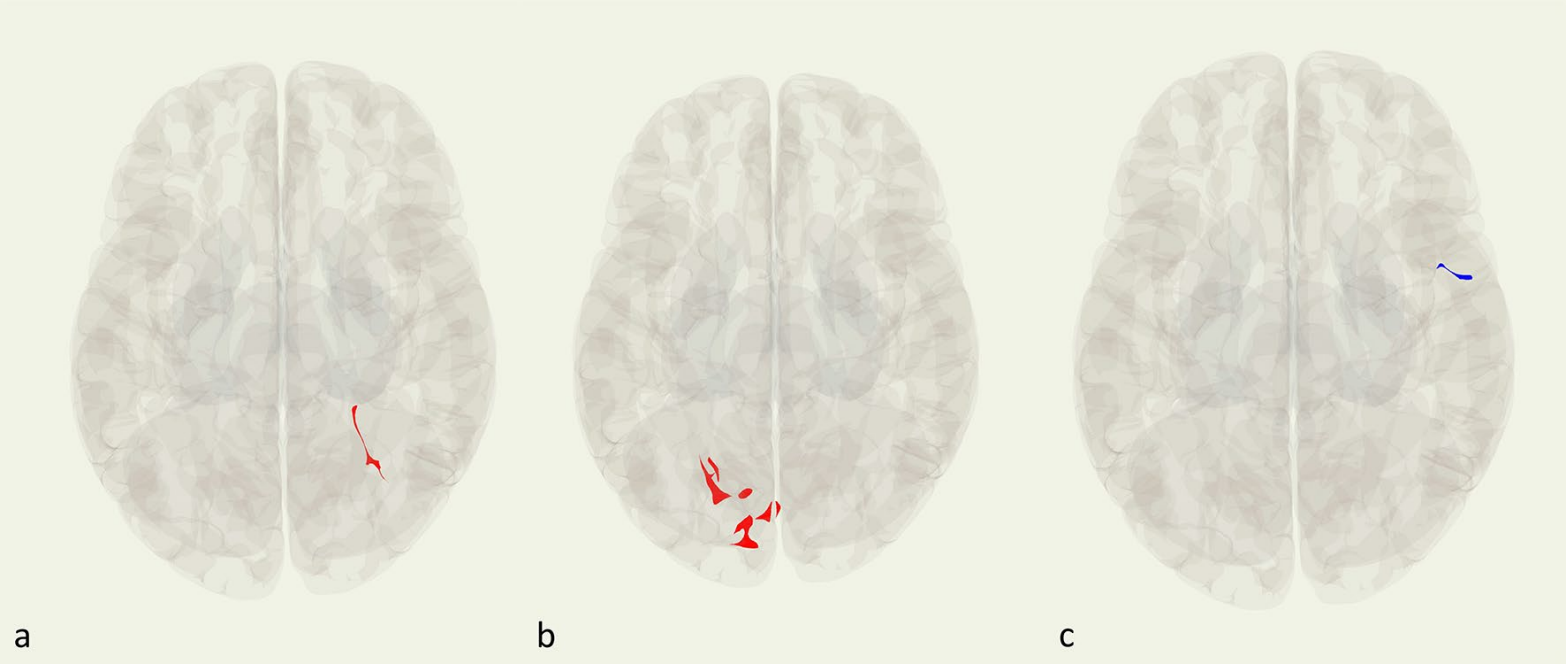
Significant cluster locations, red indicates increased resting state functional connectivity and blue indicates decreased resting state functional connectivity. (**a**) Increased resting state functional connectivity from the left lateral prefrontal cortex to the right fusiform gyrus. (**b**) Increased resting state functional connectivity from the right lateral prefrontal cortex to the left lingual gyrus, left fusiform gyrus, left intracalcarine cortex, and left supracalcarine cortex. (**c**) Decreased resting state functional connectivity from the left anterior insula to the right precentral gyrus.

Findings from regression models are reported in Table 4. Out of the 5 seeds examined, 3 had significant clusters when comparing participants who did develop a new internalizing disorder at follow-up to those who did not. Adjusting for age, sex, baseline internalizing symptoms, and internalizing disorder history, youth who experienced a new internalizing disorder onset over the follow-up period showed increased left LPFC (CCN seed) functional connectivity to the right posterior division of the temporal fusiform cortex (Fig. 2a). Youth who experienced a new internalizing disorder onset also show increased right LPFC (CCN seed) functional connectivity to 5 clusters centering around the left lingual gyrus, the left occipital fusiform gyrus, the right calcarine cortex (cluster included intracalcarine cortex, and the supracalcarine cortex), the left intracalcarine cortex, and the left supracalcarine cortex (Fig. 2b). Youth who experienced a new internalizing disorder onset additionally showed decreased functional connectivity of the left anterior insula (SN seed) with a cluster centered in the right precentral gyrus (Fig. 2c).

**Table 4:** Regressions predicting new onset of internalizing disorders adjusting for baseline internalizing symptoms.

Seed region	MNI peak coordinates (x, y, z)	Brain region	Cluster size	Increase/decrease connectivity	Cluster level *p* (FDRc)
Left LPFC	(24, − 40, − 18)	Right fusiform cortex	54	Increase	0.0035
Right LPFC	(− 10, − 82, 00)	Left lingual gyrus	104	Increase	0.00001
	(− 20, − 76, − 18)	Left fusiform gyrus	86	Increase	0.000034
	(2, − 78, 8)	Right intracalcarine cortex	46	Increase	0.003102
	(− 8, − 72, 12)	Left intracalcarine cortex	43	Increase	0.003538
	(− 22, − 60, 14)	Left supracalcarine cortex	37	Increase	0.006745
Left anterior insula	(52, 00, 46)	Right precentral gyrus	45	Decrease	0.008637

*MNI* Montreal Neurological Institute, *FDR* false discovery rate, *LPFC* lateral prefrontal cortex.

Resting state functional connectivity from the PCC and right AI seeds was not significantly different between participants who developed a new disorder onset and those who did not.

### Predicting first episode diagnosable onset of DSM-V internalizing disorder

**Figure 3:**
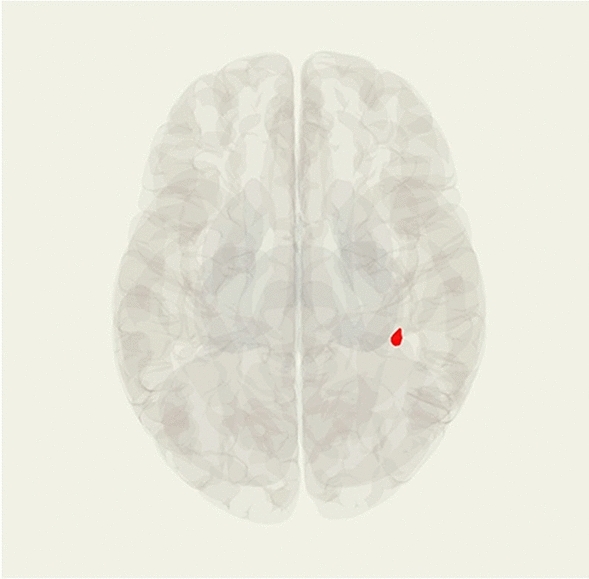
Significant cluster locations, red indicates increased resting state functional connectivity. Increased resting state functional connectivity from the right anterior insula to the right postcentral gyrus.

When excluding participants who had a lifetime history of depression or anxiety 1 of the 5 seeds had a significant cluster when comparing participants who developed a first lifetime onset compared to those who did not. Adjusting for age, sex, and baseline internalizing symptoms, youth who experienced a first episode internalizing disorder onset over the follow-up period showed increased right AI (SN seed) functional connectivity to the right postcentral gyrus (Table 5, Fig. 3).

**Table 5:** Regressions predicting first onset of internalizing disorders adjusting for baseline internalizing symptoms.

Seed region	MNI peak coordinates (x, y, z)	Brain region	Cluster size	Increase/decrease connectivity	Cluster level *p* (FDRc)
Right anterior insula	(38, − 34, 48)	Right postcentral gyrus	45	Increase	0.000763

*MNI* Montreal Neurological Institute, *FDR* false discovery rate.

Resting state functional connectivity from the PCC, bilateral LPFC and left AI seeds was not significantly different between participants who developed a first episode disorder onset and those who did not.

## Discussion

Adolescence is a period of high neuroplasticity and vulnerability to increased onset of mental illness that can become chronic in the developing brain^50^. Identifying physiological indicators of depression and anxiety before disorder onset in adolescence will inform knowledge of the pathophysiology of these disorders and may be key to early identification and prevention of these disorders that make up a significant portion of economic burden worldwide, particularly in adolescents^50^.

This study found that the increased functional connectivity between the LPFC and regions of the visual network at baseline, including the fusiform gyrus, lingual gyrus, and areas of the calcarine cortex, differentiated participants who developed a new internalizing disorder onset at follow-up compared to those who did not. The fusiform gyrus has been implicated in task-based fMRI studies, with some finding increased activity in the region during a stress-inducing task in healthy adolescents and others finding decreased connectivity between the fusiform and cognitive control regions in MDD and SAD patients compared to controls^51–53^. Structural studies looking at cortical thickness, surface, area and gyrification have also implicated the fusiform cortex, showing an overall decrease in structural metrics in MDD, people with cognitive vulnerability to depression, and high-risk offspring^54–58^. In addition to being key areas of the visual network the fusiform, lingual, and calcarine are also involved in attention, emotion processing, social cognition, and memory, often working to facilitate multi-modal integration and inhibitory control^45,59–62^.

While this study initially focused on seed regions representing hubs of the CCN, DMN, and SN, we found more distributed results in line with current research on the role of sensory regions in depression and anxiety. For example, patients with treatment-resistant depression had lower functional connectivity between the left and right fusiform than those with treatment-responsive depression, and lower functional connectivity between the bilateral calcarine cortices than both treatment-responsive and healthy controls^59^. In further analysis the RSFC between the bilateral calcarine cortices discriminated between depressed and non-depressed subjects^59^. In adults with GAD a study found decreased resting-state connectivity between the right DLPFC, and the left lingual gyrus compared to healthy controls^63^, while another found adolescents with MDD had higher positive functional connectivity between the left DLPFC and left lingual gyrus than controls^64^. Despite ample evidence of both the LPFC and these visual regions in depression and anxiety, few studies have found a behavioural correlation explaining the potential role of functional connectivity between these regions in depression and anxiety. In healthy adults resting state activation of the lingual gyrus was positively correlated with attachment avoidance scores and the study proposed it was related to the role of the lingual gyrus in memory formation and retrieval of representations of others^65^. A study looking at resting state networks in participants who reported experiencing versus high versus low stress found that the high stress participants had increased activity in the visual network at rest, reflecting the hypervigilance and alertness often seen stressed individuals^66^. A graph theory-based study found that patients with SAD exhibited increased connectivity between the frontolimbic and sensory/perceptual processing circuits and argued that it represented top-down control over enhanced emotional perception in SAD^67^. Together these studies indicate that even at rest the visual system is involved in depression and anxiety potentially due to its roles in attention and memory. However, the current study cannot delineate whether the LPFC is influencing the visual system or vice versa.

This study also found decreased functional connectivity between the left anterior insula and the right precentral gyrus predicting new onsets. The anterior insula is an important resting state network hub and is a key node in the SN^30^. Functional connectivity studies found that patients with affective disorders and their relatives had decreased activation in the insula at rest^68–73^ and during social and emotional tasks^74,75^. Gray matter reductions and hypogyrification have also been found in the insula of MDD patients and relatives^76–79^. The precentral gyrus is home to the primary motor cortex and is involved in voluntary movement, response inhibition, emotion driven action, working memory, and Theory of Mind^58,80^. Structural studies of MDD and high-risk subjects showed cortical thinning of the precentral gyrus and reduced gray matter volume^56–58,81,82^. In adult MDD, decreased resting state functional connectivity has been found in the precentral gyrus^72,77,83–87^ as well as in response to reward^88^. MDD patients with a history of suicide attempt and teens with MDD and high suicidal ideation (compared with MDD with low suicidal ideation and healthy controls) had decreased precentral gyrus activation in a verbal fluency and social task respectively^74,89^. This association of altered precentral gyrus activity and suicidality is believed to be related to the precentral gyri’s role in response inhibition and impulsivity^74,89,90^. Similarly altered precentral gyrus activation in GAD patients in response to emotional distractors during a working memory task could be related to abnormal response inhibition as well as the working memory aspect of the precentral gyrus^91^. Taken together this indicates a deficit in detecting and responding to salient information in internalizing disorders which may related to future onsets of internalizing disorders.

When exclusively examining first episode onsets we found increased RSFC between the right AI and the right postcentral gyrus. The postcentral gyrus is home to the somatosensory cortex which as been implicated in pain processing, empathy, emotional stimulus evaluation, emotion generation, and emotional regulation (see Kropf for review^92^). This increased connectivity was surprising as studies in MDD and GAD have found decreased postcentral gyrus activity during rest, and a functional connectivity study found decreased connectivity between the AI and the postcentral gyrus in somatic depression^70,93–97^. It is important to note only 2 of these studies looked at first episode and none of these studies focused on adolescents^70,85^. In bipolar disorder, two studies have found increased RSFC of the AI to the postcentral gyrus but again these were done in adults with a previous history of bipolar disorder^98,99^. This increased connectivity between the right AI and the right postcentral gyrus may represent a unique marker for first onsets of internalizing disorders in high-risk adolescents. This connectivity may influence the emotional dysregulation seen in depression and anxiety, as the AI and the somatosensory cortex play a role in stimuli salience and regulation of emotion^8,10,30,92^.

Identifying RSFC anomalies as a potential biomarker for the development of depression and anxiety may facilitate early identification of youth at risk as well as interventions to prevent first lifetime episodes of these disorders. These can include behavioral therapies as there is some evidence that they can influence network connectivity, as well as transcranial magnetic stimulation (TMS) therapies that may increase activity in underactive brain regions or inhibit overactive areas involved in the CCN^16,100^.

Our study had several strengths, such as a longitudinal design and a risk-enriched population. Our study also had limitations, such as the lack of a formal control or low-risk group to act as a comparison. While this is not necessary to test our aims as participants who did not develop a diagnosable onset act as a comparison group against those who did develop an internalizing disorder, it is unknown whether results would generalize to youth without a family history of internalizing disorders. Our sample size was also modest, and while a sufficient number of youth developed internalizing disorders for predictive purposes, we may have been underpowered to detect smaller effects, such as disorder specific alterations. Moreover, the modest number of youth with an onset of a disorder may not generalize to other populations. We were also underpowered to examine whether baseline functional connectivity predicts time to episode onset, which is likely an important future direction in understanding neural vulnerability to internalizing disorders.

This research expands the current literature on longitudinal research in adolescents at high familial risk for developing depressive or anxiety disorders. Despite adolescence being a critical period for the development of anxiety and depression, particularly in youth with a family history of the disorders, there is limited longitudinal research in the area^5^. This study found that increased connectivity between the CCN and the visual network, and decreased connectivity between the left SN and the motor cortex may represent a risk factor for developing a new onset of a depressive or anxiety disorder. By comparison, increased connectivity between the right SN and the somatosensory cortex emerged as a pre-morbid risk factor for first onsets of depression or anxiety. Results have implications for our understanding of the pathophysiology of internalizing disorders, identifying those at risk for an internalizing disorder in high-risk populations, and identifying targets for treatment and prevention.

## Supplementary Information

Supplementary Information.

## Data Availability

All data reported in this study are available via contacting the corresponding author.
