# Supplementary material for: Resting state functional connectivity as a marker of internalizing disorder onset in high-risk youth
Source: Sci Rep. 2022 Dec 9;12:21337. doi: 10.1038/s41598-022-25805-y (PMC9734132; doi:10.1038/s41598-022-25805-y)
Supplement: Supplementary file 1 — Supplementary Information. [file 41598_2022_25805_MOESM1_ESM.pdf]

Resting State Functional Connectivity as a Marker of Internalizing Disorder Onset in High-Risk Youth: Supplementary Materials

McKinley Pawlak*[1,2,3], Signe Bray[1,2,3,4,5,6], & Daniel C. Kopala-Sibley[1,2,3,7]

[1]Hotchkiss Brain Institute, University of Calgary, Calgary, AB, Canada [2]Alberta Children Hospital Research Institute (ACHRI), University of Calgary, Calgary, AB, Canada [3]Mathison Centre for Mental Health Research and Education, University of Calgary, Calgary, AB, Canada [4]Child and Adolescent Imaging Research (CAIR) Program, University of Calgary, Calgary, AB, Canada [5]Department of Radiology, University of Calgary, Calgary, AB, Canada [6]Department of Pediatrics, University of Calgary, Calgary, AB, Canada. [7]Department of Psychiatry, University of Calgary, Calgary, AB, Canada. * Correspondence should be addressed to mckinley.pawlak@ucalgary.ca.

Supplementary Figure 1.

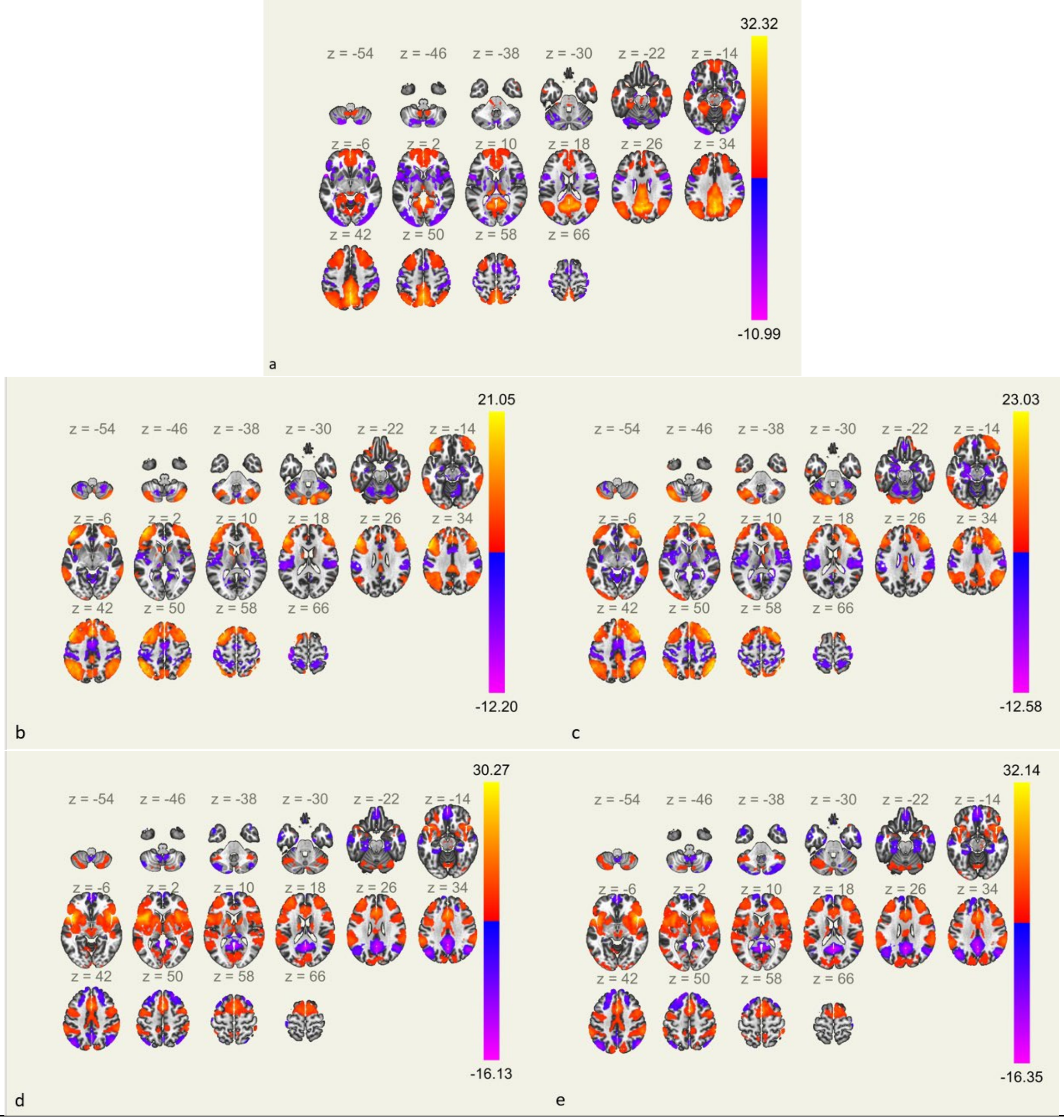

Seed to voxel connectivity maps of chosen network seeds, showing connection to the other regions of representative network, peak threshold p < .001 family wise error corrected. Positive connectivity is in warm colours, orange, yellow, red, and negative connectivity is in cool colours, purple and blue. a) The default mode network (DMN) seed, the posterior cingulate cortex, showing positive connectivity to other DMN regions and expected negative connectivity to cognitive control network regions, b and c) the cognitive control network (CCN) seeds, the bilateral lateral prefrontal cortices, showing positive connectivity to other CCN regions and expected negative connectivity to DMN regions, d and e) the salience network (SN) seeds, the bilateral anterior insula, showing positive connectivity to other SN regions.

Supplementary Table 1. Posterior Cingulate Cortex Seed Connectivity

Seed Region	MNI Peak Coordinates (x, y, z)	Brain Region	Cluster Size	Connectivity	Cluster Level *p* (FWEc)	Peak Level *p* (FWEc)
PCC	02, -66, 30	Precuneus	29608	Increase	≤ .0001	≤ .0001
	-54, 12, -06	Bilateral Precentral Gyrus	28396	Decrease	≤ .0001	≤ .0001
	02, 54, -08	Bilateral Frontal Pole	7769	Increase	≤ .0001	≤ .0001
	22, 34, 36	Right Middle Frontal Gyrus	3721	Increase	≤ .0001	≤ .0001
	-24, 28, 40	Left Middle Frontal Gyrus	3596	Increase	≤ .0001	≤ .0001
	58, -08, -22	Right Posterior Middle Temporal Gyrus	1004	Increase	≤ .0001	≤ .0001
	10, -50, -52	Bilateral Cerebellum 9	727	Increase	≤ .0001	≤ .0001
	-56, -10, -18	Left Posterior Middle Temporal Gyrus	655	Increase	≤ .0001	≤ .0001
	-32, 10, -18	Left Insular Cortex	138	Increase	≤ .0001	≤ .0001
	34, 12, -18	Right Temporal Pole	108	Increase	≤ .0001	≤ .0001
	-16, -16, -20	Left Hippocampus	89	Decrease	.000275	≤ .0001

Abbreviation: MNI, Montreal Neurological Institute; FDR, False Discovery Rate; PCC, posterior cingulate cortex

Supplementary Table 2. Left Lateral Prefrontal Cortex Seed Connectivity

Seed Region	MNI Peak Coordinates (x, y, z)	Brain Region	Cluster Size	Connectivity	Cluster Level *p* (FWEc)	Peak Level *p* (FWEc)
Left LPFC	-50, 24, 32	Bilateral Frontal Pole	26965	Increase	≤ .0001	≤ .0001
	-30, -18, 74	Bilateral Postcentral Gyrus	14898	Decrease	≤ .0001	≤ .0001
	-44, -58, 54	Left Superior Lateral Occipital Cortex	6250	Increase	≤ .0001	≤ .0001
	32, -64, -34	Right Cerebellum 2	6019	Increase	≤ .0001	≤ .0001
	26, -56, -24	Bilateral Cerebellum 6	5351	Decrease	≤ .0001	≤ .0001
	42, -58, 54	Right Superior Lateral Occipital Cortex	3846	Increase	≤ .0001	≤ .0001
	-58, -52, -10	Left Posterior Middle Temporal Gyrus	1626	Increase	≤ .0001	≤ .0001
	66, -36, -10	Right Posterior Middle Temporal Gyrus	1323	Increase	≤ .0001	≤ .0001
	00, -32, 36	Cingulate Gyrus	885	Increase	≤ .0001	≤ .0001
	26, -52, -54	Right Cerebellum 8	622	Decrease	≤ .0001	≤ .0001
	-28, -56, -54	Left Cerebellum 8	486	Decrease	≤ .0001	≤ .0001
	-12, 04, 12	Left Caudate	408	Increase	≤ .0001	≤ .0001
	14, 06, 10	Right Caudate	239	Increase	≤ .0001	≤ .0001
	30, 20, -06	Right Insular Cortex	178	Increase	≤ .0001	≤ .0001
	-12, -24, 00	Left Thalamus	101	Decrease	≤ .0001	≤ .0001

Abbreviation: MNI, Montreal Neurological Institute; FDR, False Discovery Rate; LPFC, lateral prefrontal cortex

Supplementary Table 3. Right Lateral Prefrontal Cortex Seed Connectivity

Seed Region	MNI Peak Coordinates (x, y, z)	Brain Region	Cluster Size	Connectivity	Cluster Level *p* (FWEc)	Peak Level *p* (FWEc)
Right LPFC	-32, -24, 74	Bilateral Postcentral Gyrus	25858	Decrease	≤ .0001	≤ .0001
	44, 30, 40	Bilateral Frontal Pole	24720	Increase	≤ .0001	≤ .0001
	-08, -82, -30	Left Cerebellum 2	13484	Increase	≤ .0001	≤ .0001
	48, -54, 52	Right Superior Lateral Occipital Cortex	8489	Increase	≤ .0001	≤ .0001
	64, -34, -10	Right Posterior Middle Temporal Gyrus	1619	Increase	≤ .0001	≤ .0001
	-04, 48, -16	Frontal Medial Cortex	985	Decrease	≤ .0001	≤ .0001
	-54, -18, -28	Left Inferior Temporal Gyrus	580	Increase	≤ .0001	≤ .0001
	52, -64, 02	Right Inferior Lateral Occipital Cortex	510	Decrease	≤ .0001	≤ .0001
	-26, -56, -52	Left Cerebellum 8	301	Decrease	≤ .0001	≤ .0001
	-10, 20, -02	Left Caudate	178	Decrease	≤ .0001	≤ .0001
	24, -12, -40	Right Anterior Parahippocampal Gyrus	145	Increase	≤ .0001	≤ .0001
	-32, 18, -06	Left Insular Cortex	127	Increase	≤ .0001	≤ .0001
	-06, -62, -56	Left Cerebellum 9	126	Increase	≤ .0001	≤ .0001
	-36, 24, 02	Left Frontal Orbital Cortex	106	Decrease	≤ .0001	≤ .0001

Abbreviation: MNI, Montreal Neurological Institute; FDR, False Discovery Rate; LPFC, lateral prefrontal cortex

Supplementary Table 4. Left Anterior Insula Seed Connectivity

Seed Region	MNI Peak Coordinates (x, y, z)	Brain Region	Cluster Size	Connectivity	Cluster Level *p* (FWEc)	Peak Level *p* (FWEc)
Left AI	-42, 08, -04	Right Frontal Pole	44697	Increase	≤ .0001	≤ .0001
	34, -54, -34	Right Cerebellum 1	6998	Increase	≤ .0001	≤ .0001
	00, -58, 20	Precuneus	5116	Decrease	≤ .0001	≤ .0001
	00, 56, -10	Frontal Medial Cortex	2298	Decrease	≤ .0001	≤ .0001
	-44, -70, 36	Left Superior Lateral Occipital Cortex	2096	Decrease	≤ .0001	≤ .0001
	46, -60, 32	Right Superior Lateral Occipital Cortex	1984	Decrease	≤ .0001	≤ .0001
	24, 32, 56	Right Middle Frontal Gyrus	1645	Decrease	≤ .0001	≤ .0001
	-22, 34, 56	Left Superior Frontal Gyrus	1487	Decrease	≤ .0001	≤ .0001
	-56, -10, -22	Left Middle Temporal Gyrus	1026	Decrease	≤ .0001	≤ .0001
	-24, -20, -22	Left Posterior Parrahippocampal Gyrus	860	Decrease	≤ .0001	≤ .0001
	-32, -56, -34	Left Cerebellum 1	727	Increase	≤ .0001	≤ .0001
	28, -34, -18	Right Posterior Parahippocampal Gyrus	678	Decrease	≤ .0001	≤ .0001
	58, -08, -24	Right Middle Temporal Gyrus	588	Decrease	≤ .0001	≤ .0001
	-04, -56, -50	Bilateral Cerebellum 9	566	Decrease	≤ .0001	≤ .0001
	-42, -70, -42	Left Cerebellum 2	480	Decrease	≤ .0001	≤ .0001
	-42, -30, 68	Left Postcentral Gyrus	377	Decrease	≤ .0001	≤ .0001
	42, -70, -38	Right Cerebellum 2	232	Decrease	≤ .0001	≤ .0001
	-10, -84, -42	Left Cerebellum 2	148	Decrease	≤ .0001	≤ .0001
	38, 10, -40	Right Temporal Pole	102	Decrease	≤ .0001	.0002

Abbreviation: MNI, Montreal Neurological Institute; FDR, False Discovery Rate; AI, anterior insula

**Supplementary Table 5.** Right Anterior Insula Seed Connectivity						
Seed Region	MNI Peak Coordinates (x, y, z)	Brain Region	Cluster Size	Connectivity	Cluster Level *p* (FWEc)	Peak Level *p* (FWEc)
Right AI	42, 14, -02	Right Frontal Pole	41373	Increase	≤ .0001	≤ .0001
	-32, -56, -34	Left Occipital Pole	8403	Increase	≤ .0001	≤ .0001
	00, -52, 28	Precuneus	5927	Decrease	≤ .0001	≤ .0001
	-02, 46, -16	Left Frontal Pole	5668	Decrease	≤ .0001	≤ .0001
	-40, -66, 32	Left Superior Lateral Occipital Cortex	2169	Decrease	≤ .0001	≤ .0001
	44, -60, 32	Right Superior Lateral Occipital Cortex	1764	Decrease	≤ .0001	≤ .0001
	22, 36, 44	Right Frontal Pole	1260	Decrease	≤ .0001	≤ .0001
	-58, -06, -22	Right Temporal Pole	1171	Decrease	≤ .0001	≤ .0001
	12, -86, -38	Right Cerebellum 2	1130	Decrease	≤ .0001	≤ .0001
	-44, -04, 46	Left Precentral Gyrus	1122	Increase	≤ .0001	≤ .0001
	-60, -16, -16	Left Temporal Pole	1082	Decrease	≤ .0001	≤ .0001
	-26, -36, -20	Left Posterior Parahippocampal Gyrus	1010	Decrease	≤ .0001	≤ .0001
	06, -52, -48	Bilateral Cerebellum 9	568	Decrease	≤ .0001	≤ .0001
	-06, -86, -38	Left Cerebellum 2	390	Decrease	≤ .0001	≤ .0001
	36, -50, -34	Right Cerebellum 1	360	Increase	≤ .0001	≤ .0001
	38, -16, 72	Right Precentral Gyrus	247	Decrease	≤ .0001	≤ .0001
	34, -56, -54	Right Cerebellum 8	213	Increase	≤ .0001	≤ .0001
	24, 50, -18	Right Frontal Pole	202	Increase	≤ .0001	.0002
	-26, 38, -16	Left Frontal Pole	93	Increase	≤ .0001	≤ .0001
	66, -04, 26	Right Postcentral Gyrus	78	Decrease	.0007	.0003

Abbreviation: MNI, Montreal Neurological Institute; FDR, False Discovery Rate; AI, anterior insula
